# Dosimetric validation of SmART-RAD Monte Carlo modelling for x-ray cabinet radiobiology irradiators

**DOI:** 10.1088/1361-6560/ad3720

**Published:** 2024-04-19

**Authors:** Mark A Hill, Nick Staut, James M Thompson, Frank Verhaegen

**Affiliations:** 1 MRC Oxford Institute for Radiation Oncology, Department of Oncology, University of Oxford, ORCRB Roosevelt Drive, Oxford OX3 7DQ, United Kingdom; 2 SmART Scientific Solutions BV, Maastricht, The Netherlands; 3 Department of Radiation Oncology (Maastro), Research Institute for Oncology & Reproduction, Maastricht University Medical Centre+, Maastricht, The Netherlands

**Keywords:** dosimetery, Monte Carlo, radiobiology, pre-clinical, x-ray

## Abstract

*Objective*. Accuracy and reproducibility in the measurement of radiation dose and associated reporting are critically important for the validity of basic and preclinical radiobiological studies performed with kilovolt x-ray radiation cabinets. This is essential to enable results of radiobiological studies to be repeated, as well as enable valid comparisons between laboratories. In addition, the commonly used single point dose value hides the 3D dose heterogeneity across the irradiated sample. This is particularly true for preclinical rodent models, and is generally difficult to measure directly. Radiation transport simulations integrated in an easy to use application could help researchers improve quality of dosimetry and reporting. *Approach*. This paper describes the use and dosimetric validation of a newly-developed Monte Carlo (MC) tool, SmART-RAD, to simulate the x-ray field in a range of standard commercial x-ray cabinet irradiators used for preclinical irradiations. Comparisons are made between simulated and experimentally determined dose distributions for a range of configurations to assess the potential use of this tool in determining dose distributions through samples, based on more readily available air-kerma calibration point measurements. *Main results*. Simulations gave very good dosimetric agreement with measured depth dose distributions in phantoms containing both water and bone equivalent materials. Good spatial and dosimetric agreement between simulated and measured dose distributions was obtained when using beam-shaping shielding. *Significance*. The MC simulations provided by SmART-RAD provide a useful tool to go from a limited number of dosimetry measurements to detailed 3D dose distributions through a non-homogeneous irradiated sample. This is particularly important when trying to determine the dose distribution in more complex geometries. The use of such a tool can improve reproducibility and dosimetry reporting in preclinical radiobiological research.

## 1. Introduction

The accuracy and reproducibility in the measurement of dose, reporting of irradiation setup, along with the need for standardisation of dosimetry between labs are critically important for the validity of basic and preclinical radiobiology studies performed with kilovolt (kV) x-ray radiation cabinets (Desrosiers *et al* 2013, Verhaegen *et al* 2018). This is essential, not only to enable results of radiobiological studies to be repeated and enable valid comparisons between laboratories, but importantly to assess the quality and limitations of preclinical data required to evaluate the translational potential of the resulting data and underlying hypotheses which ultimately facilitate the development of clinical trials (Liu *et al* 2013). Currently many articles lack dosimetry data and an appropriate description of the irradiation setup used. This information is required for the study to be reproducible, interpretable and comparable. Highly cited journals and articles are systematically more likely to be lacking these important details (Draeger *et al* 2020). In addition, large dose discrepancies have also been reported for surveys across a number of laboratories (e.g. Pedersen *et al* 2016) highlighting the need for standardised protocols, measurements traceable to national standards and regular inter-laboratory audits.

Accurate photon dosimetry is not straightforward as radiation dose is dependent on a number of factors which may affect not only the absolute dose delivered but also the resulting 3D dose distribution through the sample. It is very rarely the case that the sample will be uniformly irradiated, but in most cases, there will be a 3D variation in dose across the sample, which can be important with respect to interpreting the results. Also, physical dose measurements may not directly correspond to the dose to the critical volume within a biological sample. These 3D dose distributions will be dependent on factors such as the x-ray energy spectra, distance from a source, field size along with the geometry and atomic composition and mass density of the sample being irradiated and any surrounding structures. In addition to attenuation of the x-ray beam, there can also be a significant contribution to dose from backscattered radiation (Chen *et al* 2019). While it may be possible to perform accurate dosimetry for specific cases often by using simplified geometries, it can be difficult to cover the wide range of geometries and samples used in practice (e.g. mouse, rat, multiple animals, cell cultures in a range of flasks/plates) along with use of custom shields, collimators etc. The low-energy x-rays produced by these kV x-ray cabinet irradiators also mean that the dose distribution and associated absorbed dose is particularly sensitive to higher atomic number materials due to the contribution of the photo-electric effect (Poirier *et al* 2020). Therefore, in animals the attenuation and associated dose deposition in bone is significantly higher and more heterogeneous (e.g. in the bone marrow) than in the surrounding material; the opposite can be observed in adipose tissue. Also, this can result in significant interface effects, for example cells grown on glass will receive a significantly higher dose for a given x-ray exposure than cells grown on plastic (Hood and Norris 1961, Furre *et al* 1999). In addition, for animal samples it is often not sufficient to use the average dose to a simplified geometry animal phantom, as the 3D dose distribution can vary significantly through the animal, while the actual dose required is that to a specific organ or volume of interest at a given position in or on the animal. This variation becomes more pronounced for lower tube potentials and softer beam filters. The only way to get a realistic description of this 3D dose distribution is through modelling radiation transport through mathematical 3D phantoms based on imagery or mathematical models (Segars *et al* 2004). When using such radiation transport simulations it is also important to consider the dose quantifiers such as dose to water in water (*D*_w,w_), dose to water in medium (*D*_w,m_) and dose to medium in medium (*D*_m,m_) (Vaniqui *et al* 2019). Physical dose measurements typically report dose to water and using these values to represent 3D dose distributions ignores the fact that certain media will receive a significantly higher or lower dose, especially in the lower energy kV x-ray irradiations, and there may also exist spectral changes with depth in the specimen. At present it is unknown if *D*_w,w_, *D*_w,m_ or *D*_m,m_ correlate best with biological response. The effect of choosing a different dose reporting method have large differences in dose for low energy kV irradiations, Monte Carlo (MC) simulations provide a means to calculate all these metrics (Verhaegen *et al* 2014).

A number of these issues are starting to be addressed with the advent of image-guided pre-clinical small animal irradiators where onboard cone beam CT imaging is used in conjunction with treatment planning software and detailed dosimetric commissioning (Brown *et al* 2022, Verhaegen *et al* 2023). In addition to delivering a known dose to a targeted volume within the specimen with single, multiple or arc collimated x-ray beams, the software will also calculate the 3D dose distribution throughout the irradiated sample (van Hoof *et al* 2013, Cho *et al* 2018). While these image-guided irradiators clearly offer great opportunities for the advancement of pre-clinical radiobiological research, the vast majority of current research in the field is still performed using standard x-ray or ^137^Cs *γ*-ray cabinets. While restricted to more simplified radiation geometries, these irradiators are significantly cheaper and generally offer a higher throughput. However, one of the big issues faced with achieving accurate and reproducible dosimetry for both *in vitro* and *in vivo* radiobiological studies with these standard x-ray cabinet irradiators is that traditionally many are sold or acquired with no dosimetry. It is then up to the users to find physics support to calibrate these machines. In the instances where manufacturers and suppliers do offer a dosimetry service, this is generally restricted to one or two configurations and is often provided in the form of air-kerma measurements for a given point in air or on the surface of a shelf, rather than dose to the sample of interest determined by direct measurements in the sample (which is often difficult) or a realistic phantom.

There are growing concerns of the potential misuse of high activity sealed sources (HASS) for malicious intentions, which has resulted in a worldwide push to ban or restrict the use of caesium high-activity sealed sources. For example, in the US there is the Office of Radiological Security Cesium Irradiator Replacement Project (CIRP), with a similar project (Operation Fieldfare) currently under discussion in the UK by the Joint Security and Resilience Centre (JSaRC) within the Home Office. As a result, there is growing interest in the use of x-ray cabinet irradiators as a replacement for existing caesium irradiators (Murphy and Kamen 2019, Barnard *et al* 2020). With a significant difference between the high-energy monoenergetic gamma-rays (662 keV) emitted by ^137^Cs source compared to the broad spectrum of much lower energy x-rays (typically up to a maximum energy of 320 keV) emitted by orthovoltage x-ray tubes, there is a need to be able to model the resulting dose distributions through samples as well as gain more insight in the dose distribution for non water-equivalent materials (e.g. adipose, bone, lung). For ^137^Cs *γ*-ray sources the Compton scattering effects dominates, so dose differences in different tissues are minimal, whereas for kV irradiators photo-electric effect will also play an important role, leading to significant tissue effects on dose.

The accuracy and reproducibility of dose delivery for preclinical and radiobiological investigations are of great importance. MC simulations and corresponding measurements specific to the experimental setup are therefore vital to achieve these goals (Zhong *et al* 2020). This paper describes the use and dosimetric validation of a newly-developed MC tool, SmART-RAD, to simulate the x-ray field in a standard commercial x-ray cabinet irradiator used for preclinical irradiations. Comparisons are made between simulated and experimentally determined dose distributions for a range of configurations in order to assess the potential use of this tool in determining dose distributions through samples, based on the more readily available air-kerma calibration measurements. Also a comparison between dose distributions of kV x-rays and ^137^Cs *γ*-rays is shown in rodent anatomy.

## 2. Methods

### 2.1. X-ray cabinet irradiations

X-ray irradiations were performed using a 320 kV x-ray cabinet (Gulmay Medical, now known as Xstrahl Ltd Walsall, UK) with a MXR-321 tube (Comet, Flamatt, Switzerland) with a 30° anode angle, 0.2 mm copper filter and inherent filtration of 3.0 mm Be. Irradiations were performed at three different conditions along with calculated half value layers (300 kV, 10 mA, HVL(Cu) = 1.1 mm; 200 kV, 12 mA, HVL(Cu) = 0.6 mm; 100 kV, 25 mA, HVL(Cu) = 0.2 mm), with samples placed on a 1 cm thick Perspex shelf at a distance of 500 mm from the x-ray focal spot. All irradiations were performed with a open field.

### 2.2. Dosimetry

Air-kerma measurements were performed in the RS320 x-ray cabinet using a NE2581 ionisation chamber (Nuclear Enterprises, Berkshire, UK) connected to a NE2570/1 Farmer Dosemeter (Nuclear Enterprises, Berkshire, UK), positioned in air 500 mm from the tube’s focal spot. Dosimetry measurements within the phantom were performed using EBT3 Gafchromic film (Ashland Advanced Materials LLC, Niagara Falls, NY) which were scanned as 48 bit RGB TIFF images at 300 dpi resolution using an Expression 10000 XL flatbed scanner (Seiko Epson, Japan) at 24 h post-irradiation. The dose was then calculated using a three-colour correction in conjunction with a calibration curve (Micke *et al* 2011). The reported uncertainty in EBT3 film is the order of 2.6%, 4.3% and 4.1% for the red, green and blue channels, respectively (Marroquin *et al* 2016).

Absolute dosimetry was performed following the AAPM Task Group 61 report (Ma *et al* 2001). This used 220 kV x-rays (0.15 mm copper filter) from a gantry mounted Comet MXR-321 x-ray tube (AGO x-ray Ltd Yeovil, UK) in a shielded room to deliver a 10 cm × 10 cm field at 1 m on to the surface of a Plastic Water^®^ LR (CIRS Norfolk VA, USA) phantom with an NE2581 ionisation chamber positioned centrally to the beam at a depth of 2 cm. Both the ionisation chamber and the associated NE570/1 Farmer Dosemeter were calibrated at the National Physical Laboratory (UK). The calibration films were also subsequently exposed at a depth of 2 cm in the Plastic Water^®^ LR phantom using the same setup.

### 2.3. Monte Carlo modelling

**Figure 1.**
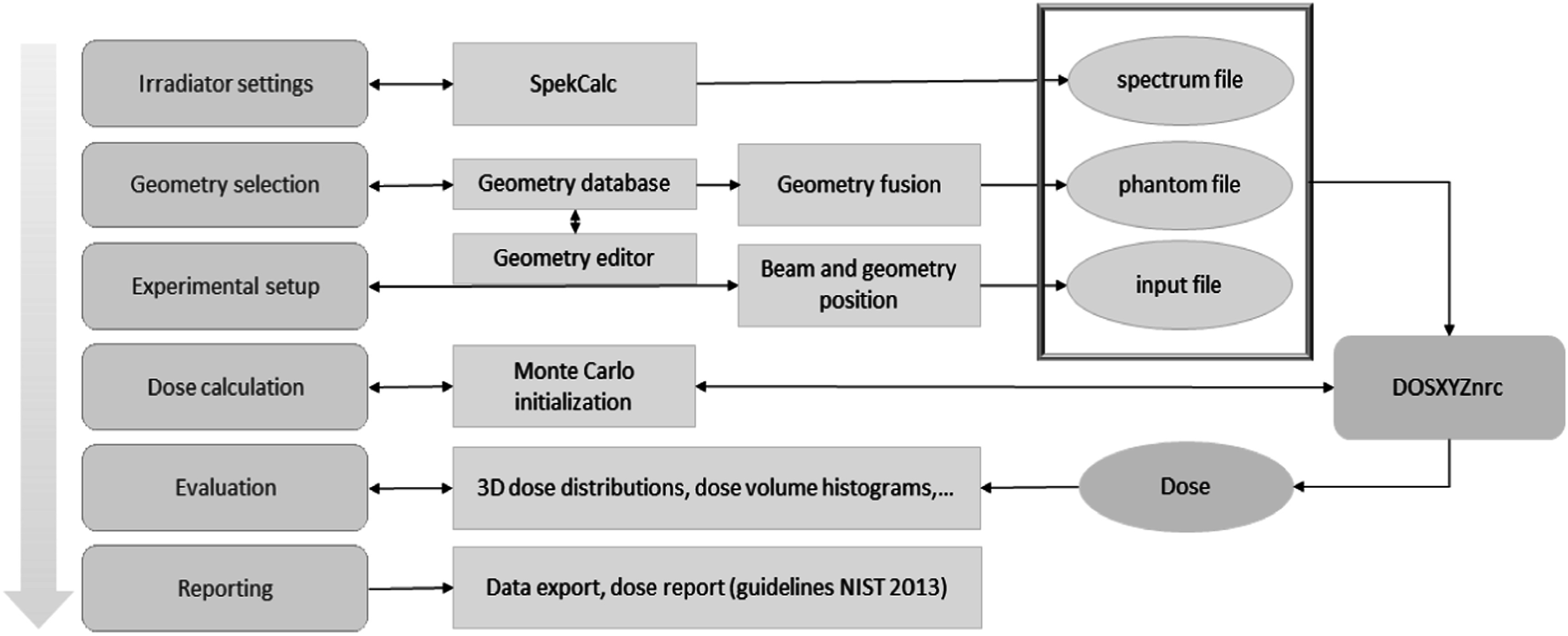
Flowchart of the SmART-RAD workflow, the blue sections each represent a module inside SmART-RAD. In these modules the user can easily modify all parameters as it is represented in the real-world setup or use it to evaluate and export the results. The orange sections represent the underlying processes. The green blocks illustrate the workflow of the Monte Carlo dose engine.

MC modelling was performed using SmART-RAD (figure 1). SmART-RAD is a tool developed in MATLAB (v9.11.0.2022996, R2021b, The MathWorks Inc., Ma) and is distributed as a compiled executable program that runs on Windows 10 or later. It provides an easy to use graphical user interface over a custom version of the DOSXYZnrc user program (Walters *et al* 2010) allowing non-MC experts to accurately calculate dose distributions for biological x-ray cabinet experiments for both simple and complex setups (Walters *et al* 2010). Expert users can modify all MC parameters inside the SmART-RAD user interface. A non-exhaustive list of default physics and variance reduction parameters can be found in table 1.

**Table 1.** Non-exhaustive list of default physics and variance reduction parameters used by SmART-RAD. Settings followed by a (*) were turned on during the slit shield modelling.

Dosxyznrc parameter	Default setting
Global ECUT	0.831
Global PCUT	0.01
Boundary crossing algorithm	PRESTA-I
Electron step algorithm	PRESTA-II
Spin effects	off
Bound compton scattering	on
Radiative compton corrections	off
Photoelectron angular sampling	off
Rayleigh scattering	on
Atomic relaxations	off*
Electron impact ionization	off*
Photon cross sections	xcom
n_split	200

Geometry definitions are handled by using a large database with over 6000 differently sized pre-generated rat and mouse models based on MOBY and ROBY mathematical phantoms (Segars *et al* 2004), as well as a module that allows modifying or creating existing geometries using Boolean combinations of geometric shapes. All geometric shapes are shown to the user in a 3D rendering of correct relative sizes to evaluate their setup as well as a 2D views of the voxelized geometry in different planes with tissue definitions.

Dose calculations are done using an analytically calculated x-ray spectrum and a collimated point source inside DOSXYZnrc. This simplified method of modelling the x-ray source is chosen to allow for a wide range of x-ray spectra as well as ^137^Cs *γ*-rays. Additionally, analytical corrections are applied to handle differences in radial dose distribution such as the additional path length through the filter for photons emitted away from the central axis. Optionally other phenomena like the anode heel effect can be corrected based on measured dose profiles.

SmART-RAD also offers a range of tools to evaluate the 3D dose distributions such as visual colour washes, dose-volume histograms and dose-volume metrics as commonly used in radiotherapy but potentially also useful for analysing radiobiology dose distributions. There is also the option to export a report containing all recommended information as found in Desrosiers *et al* (2013).

The atomic compositions assumed for Plastic Water^®^ LR and RB2 bone (Phoenix Dosimetry Ltd Berkshire, UK) equivalent materials assumed for the MC simulations are presented in table 2. The density of RB2 bone was taken to be 1.310 g cm^−3^, determined by measuring the dimensions and weight of the slabs used. This is consistent with densities reported in the literature which ranged from 1.26 to 1.40 g cm^−3^ (Poludniowski *et al* 2011, Leeds Test Objects Ltd 2014, Richmond *et al* 2021). In these calculations EBT3 film was modelled as Plastic Water LR with a thickness of 0.278 mm. Lead used for shaping beams was assumed to be 100% pure (*A* = 207.19) with a density of 11.35 g cm ^−3^.

**Table 2.** Atomic composition (mass fraction) of plastic water LR and RB2 bone used for Monte Carlo simulations (Leeds Test Objects Ltd 2014, Schoenfeld *et al* 2015).

Element	H (*A* = 1.008)	C (*A* = 12.011)	N (*A* = 14.007)	O (*A* = 15.999)	Mg (*A* = 24.305)	Cl (*A* = 35.453)	Ca (*A* = 40.080)
Plastic water LR	7.91%	53.62%	1.74%	27.21%	9.29%	0.23%	—
RB2 bone equivalent	5.71%	50.58%	1.67%	28.20%	—	0.10%	13.74%

### 2.4. Depth dose measurements and Monte Carlo dose conversion factor

DOSXYZnrc simulations require a conversion factor to translate their dose units, Gy per primary particle, into an absolute Gy value that matches the machine output in mAs. Usually a conversion factor is determined experimentally for each individual spectrum (Popescu *et al* 2005). To allow dose calculations over a wide range of spectra, SmART-RAD uses SpekCalc, an analytical model to calculate x-ray spectra (Poludniowski 2007, Poludniowski and Evans 2007, Poludniowski *et al* 2009). Typically a fixed conversion factor for a single spectrum is determined based on measurements. SmART-RAD provides the option to replace this conversion factor with a scaling factor for the number of photons in the spectrum resulting from SpekCalc. This allows the use of previously uncalibrated x-ray spectra, but introduces additional uncertainty for these uncalibrated spectra. If this scaling factor is used, the new conversion factor is determined based on one or more reference simulations at one or more arbitrary energies. These comparisons are used to determine a scaling factor using equation (1)

\begin{eqnarray*}{F}_{\mathrm{cal}}={S}_{\mathrm{ref}}* \frac{\int {N}_{\mathrm{beam}}\left(E\right){dE}}{\int {N}_{\mathrm{ref}}\left(E\right){dE}}* \frac{{A}_{\mathrm{beam}}* {{d}_{\mathrm{ref}}}^{2}}{{A}_{\mathrm{ref}}* {{d}_{\mathrm{beam}}}^{2}},\end{eqnarray*}

*F*_cal_ = conversion factor (particles/mAs) used to convert the MC simulation (Gy/particle) to absolute dose rate (Gy/mAs) *A* = beam area at isocenter *d* = source to isocenter distance (SID) *N* = number of photons in each energy bin of the calculated energy spectrum *S*_ref_ = experimentally determined scaling factor for reference spectrum unique to each machine.

Where *A* and *d* are setup dependent values used in the MC simulation (isocenter is the DOSXYZnrc definition of isocenter which SmART-RAD places at the surface in the middle of the shelf).

Calculated depth dose distributions were compared with dose measurements for an uncollimated x-ray field at the three different voltages to validate the simulation output and scaling factor.

**Figure 2.**
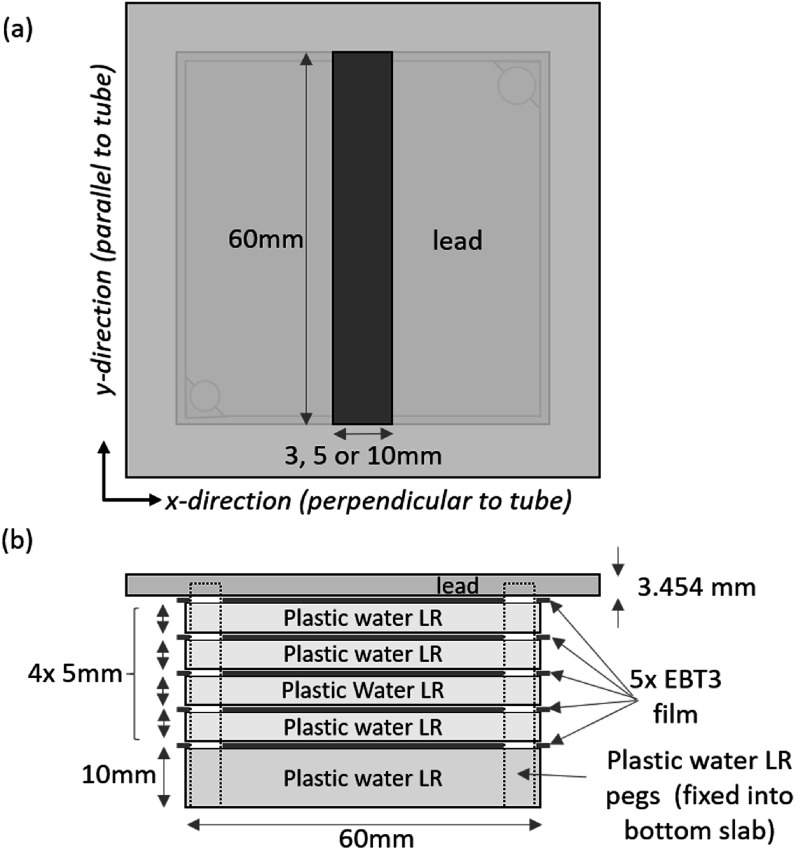
Schematic representation of the plastic water depth dose setup, used with and without the lead shield (with a slit of either 3, 5 or 10 mm wide). For depth dose measurements, no lead shield was used and in addition to plastic water (each 5.04 mm thick), measurements were also made using four 5.03 mm thick slabs of RB2 bone equivalent material. The Perspex shelf is not shown in this figure.

Simulations were performed by modelling the known materials used in the measurements. The film layers were modelled as H_2_O; this material was chosen as the film was calibrated to give dose to water and DOSXYZnrc gives dose to medium in medium. All simulations except radial distribution were performed with a sufficient number of particles to have <1% statistical uncertainty in the region with >50% of max dose at the depths where film was modelled, statistical analysis is based on a history by history method. This simulation approach was used for modelling of all other measurements. To reduce computation time the radial dose simulation was run to <2% uncertainty. Measurements were performed using a tissue equivalent phantom (figure 2) made of either four slabs (60 mm × 60 mm) of Plastic Water LR (each with a measured thickness each of 5.04 mm) or four similar slabs of RB2 (average bone, measured thickness each of 5.03 mm), separated by five EBT3 films. These were located in a holder with a 10 mm thick base of Plastic Water LR, positioned centrally on the 10 mm thick Perspex shelf (not depicted) 500 mm from the x-ray focal spot as illustrated in figure 2

### 2.5. Slit irradiations

In addition to the standard depth dose distribution using plastic water, similar irradiations were also performed with an addition lead shield (80 mm × 80 mm × 3.454 mm) placed on top of the phantom. This lead shield included a slit positioned centrally, which went across the length of the phantom and had a width of 3 mm, 5 mm or 10 mm as shown in figure 2. The phantom was either positioned centrally to the x-ray beam as before or offset from the centre by 100 mm (as it is common for multiple mice to be irradiated after placing at the same radial distance from the centre of the beam, using lead to partially shield).

### 2.6. Radial dose distribution and heel effect

The variation in radial dose across the shelf was determined using fifteen 20 mm wide strips of EBT3 film lined up side by side at 20 mm intervals across the Perspex shelf positioned underneath a 5 mm thick slab of plastic water. Irradiations were performed for 300 kV x-rays with films lined up either perpendicular to the tube (*x*-direction) or parallel to the tube (*y*-direction), with positive positions corresponding to the direction of the anode across the centre of the shelf.

The simulations assume a single large film, with the average dose calculated for a 20 mm×20 mm region of interest, to represent the individual films. While SmART-RAD takes the solid angle binning and inverse square law into account, the source is modelled as a truncated isotropically emitting point source with an energy spectrum. To compensate for this simplification the resulting dose distribution in Gy/particle is corrected by the number of particles expected to reach a certain point. This correction can be based per spectrum on a set of measurements to account for additional path length through the filter and anode heel effect. Measurement-based corrections were not used on any of the simulations in this paper as they would likely be spectrum dependent and the aim is to demonstrate the capabilities outside the calibrated spectrum. If no measurements are available an estimation for the additional path length for non-perpendicular rays through the filter is applied. The thickness of filter traversed for each voxel position, *t*(*x, y, z*), can be calculated as shown in equations (2)–(3)

\begin{eqnarray*}\varphi (x,y,z)={\tan }^{-1}\frac{\sqrt{{x}^{2}+{y}^{2}}}{\mathrm{SID}+z}\end{eqnarray*}

\begin{eqnarray*}t(x,y,z)=\frac{{t}_{\mathrm{filter}}}{\cos \varphi (x,y,z)},\end{eqnarray*}

where SID represent the x-ray source to shelf distance and *t*_filter_ is the thickness of the filter. The real number of photons seen by a voxel at a specific position, *N*_trans_(*x*, *y*, *z*), can be calculated using equation (4):

\begin{eqnarray*}{N}_{\mathrm{trans}}(x,y,z)=\displaystyle \sum _{i}^{n}{N}_{i}\times {e}^{-(t\left(x,y,z\right)-{t}_{\mathrm{filter}})\times {\mu }_{\mathrm{att},i}}\end{eqnarray*}

where *N*_*i*_ represents the number of photons behind the filter for a perpendicular ray (as calculated by SpekCalc) for a given energy bin of the x-ray spectrum, *μ*_att*,i*_ is the filter material linear mass attenuation coefficient for the given photon energy E_i_ multiplied by the density of the filter. The change in spectrum introduced by the minimal amount of copper is ignored and therefore the ratio of particles can be used directly to scale the dose distribution for any position (*x*, *y*, *z*).

## 3. Results

### 3.1. Air kerma measurements

A comparison between the measured values of air kerma (as described in section 2.2) and the SpekCalc analytically derived values are given in table 3 for x-ray energies of 100 kV, 200 kV and 300 kV. The calculated ratio of the measured to analytic SpekCalc value is relatively consistent across the three energies, with a mean value of 1.32 ± 0.05. This ratio is used in subsequent calculations to scale the output of the SmART-RAD simulation to determine dose.

**Table 3.** Comparison measured air kerma and analytically determined air kerma.

Energy (kV)	Dose rate (Gy/min) for 10 mA at 500 mm distance		Ratio (measured/analytical)
	Measurement	Analytical value	
300	1.560 ± 0.013	1.22	1.27
200	0.715 ± 0.003	0.53	1.34
100	0.162 ± 0.002	0.12	1.35
		Mean	1.32
		Standard deviation	0.05 (4.1%)

### 3.2. Depth dose distributions

**Figure 3.**
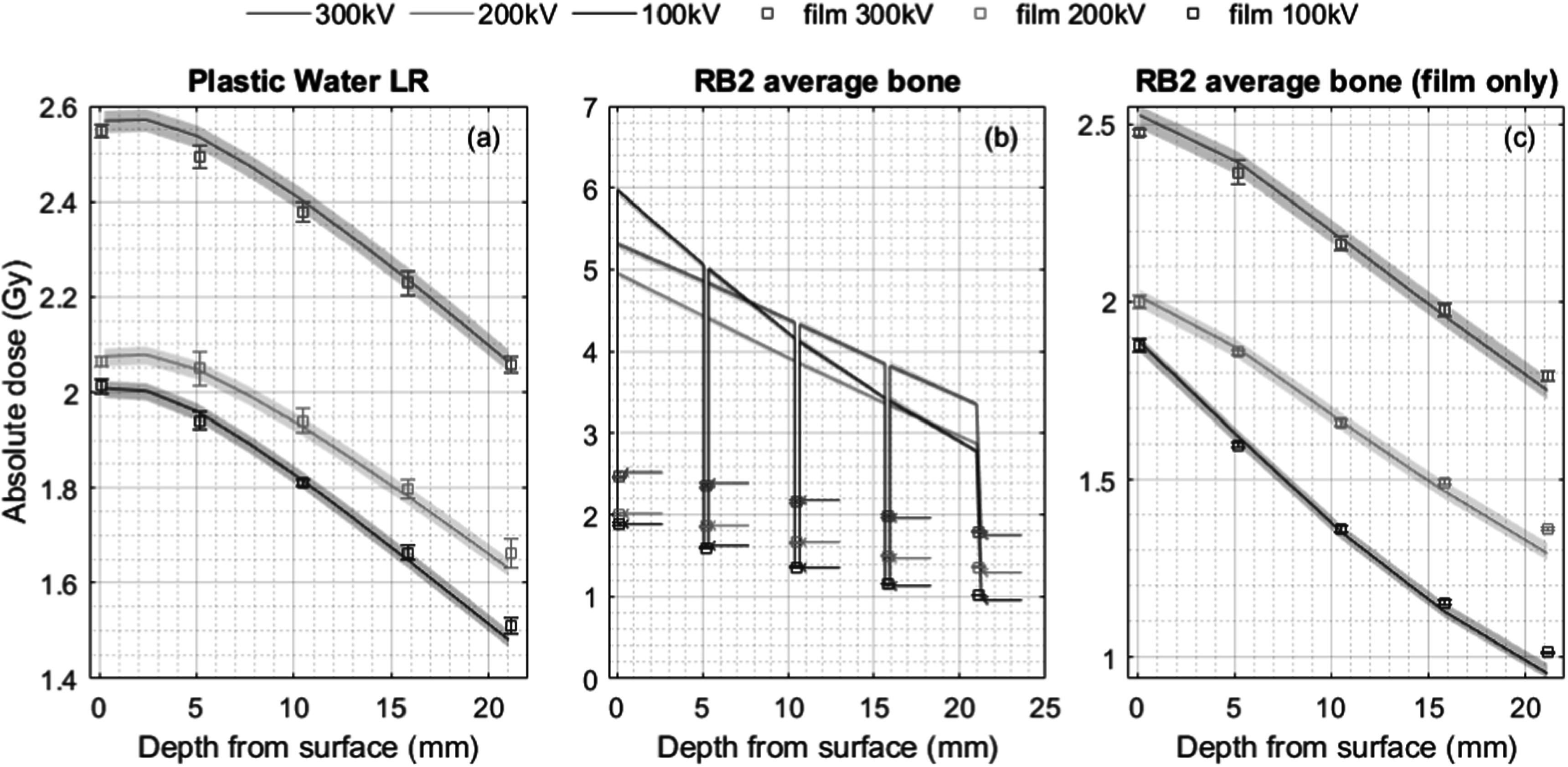
Comparison of depth dose measurements and SmART-RAD simulations in: (a) Plastic Water^®^ phantom, (b) RB2 bone equivalent phantom and (c) the RB2 phantom but only plotting the simulated values in water. The total charge delivered was 600, 1080 and 4500 mAs for the 300 kV, 200 kV and 100 kV measurements, respectively. SmART-RAD reports dose to medium in medium resulting in a much higher dose in the RB2 medium, therefore plot (b) has arrows indicating the dose to the simulated water film compared to the film measurements of dose to water (symbols). The shaded regions show the simulation uncertainty.

Comparison of the measured absolute depth dose distribution and the corresponding simulated values for the three different x-ray energies are shown in figure 3(a) for the plastic water phantom (values are tabulated in table S1) and figure 3(b) for the RB2 bone equivalent phantom (values tabulated in table S2). The simulations for Plastic Water^®^ in particular show very good absolute agreement with measurements with a maximum deviation of less than 2%. The simulation of film (water) dose sandwiched in the RB2 bone phantom also gives good agreement, with a mean deviation of 1.8% and only 2 outliers above 5% up to 6.1%. These values are within acceptable margins for preclinical research, although no official guidelines exist, 5% is often quoted as the required accuracy for clinical dose delivery to a target volume (IAEA, 2000).

### 3.3. Slit experiments

**Figure 4.**
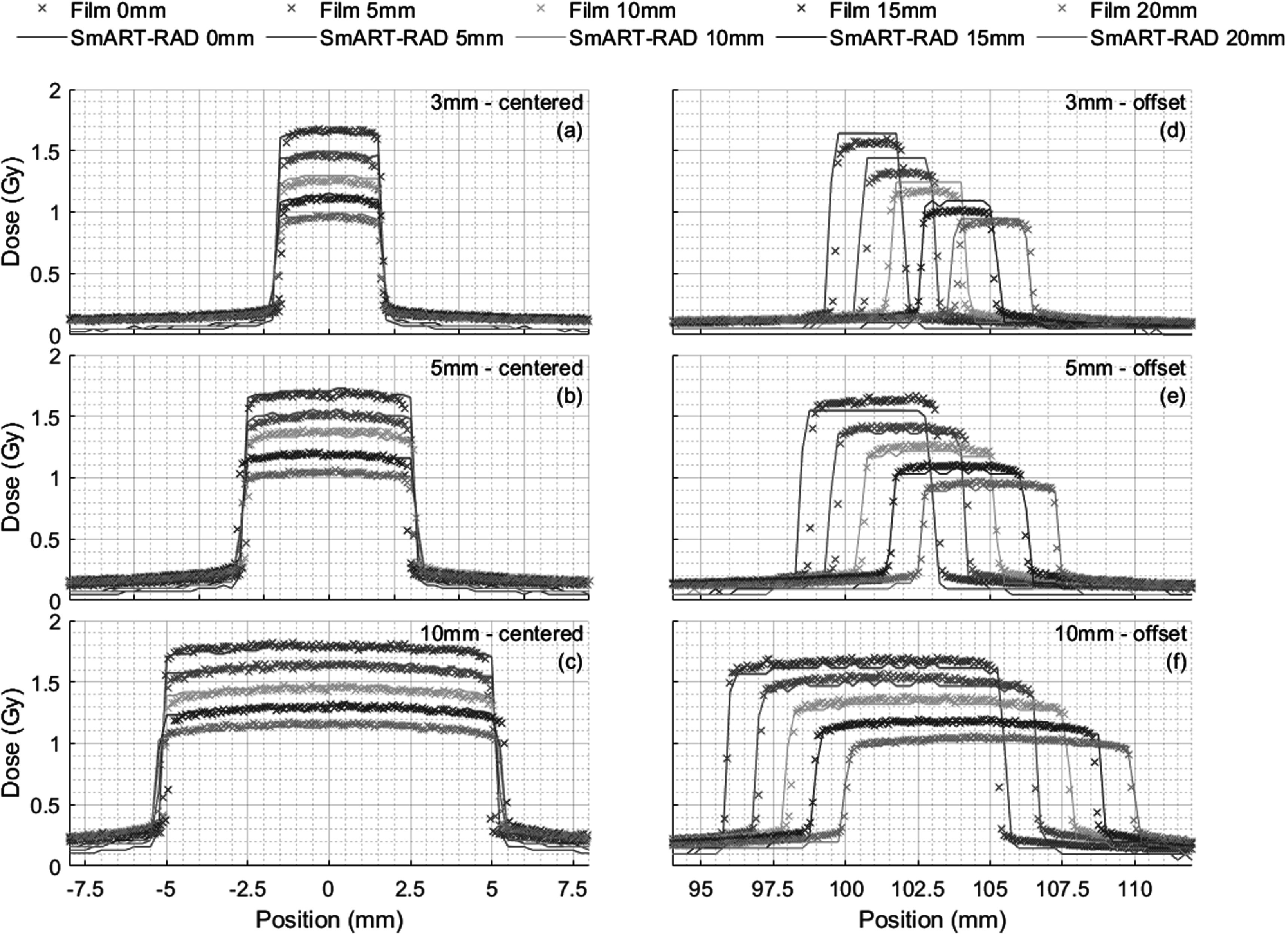
Comparison of measured and simulated central axis dose profiles as a function of depth in the phantom for 100 kV x-rays with 0.2 mm Cu filtration used in conjunction with the 3 mm (a), (e), 5 mm (b), (f) and 10 mm (c), (g) slits positioned either centrally to the beam or offset by 100 mm. A tube current of 25 mA and total exposure time of 180 s was used to irradiate each sample.

A comparison between the measured and simulated lateral dose profiles as a function of depth in the phantom used in conjunction with the 3 mm, 5 mm and 10 mm slits positioned either centrally to the beam or offset by 100 mm for 100 kV x-rays is shown in figure 4 and for 300 kV x-rays in figure S1. The dose metrics in tables S3–6 are mean values over the voxels with at least 80% of the maximum dose, to average out the noise. In general, there is good dosimetric agreement within 3.5% in the irradiated area for all centred slit exposures but SmART-RAD calculations showed a lower dose in the shielded areas of on average 5% less than the maximum dose compare to the measurements. The exposures within the open slit with 100 mm offset showed higher dosimetric disagreement on average 3.5% difference but with outliers up to 8.6%, with the simulations overestimating the dose. This is probably coupled with the difference in radial dose drop-off (see next section). The spatial agreement at full width half max (FWHM) is good, within 0.5 mm for all measurements and on average the deviation is within 0.2 mm. The simulations tend to overestimate the beam size compared to films. This could be an effect of the voxelization of the geometry as the voxel spacing was 0.25 mm.

### 3.4. Radial dose distribution and heel effect

**Figure 5.**
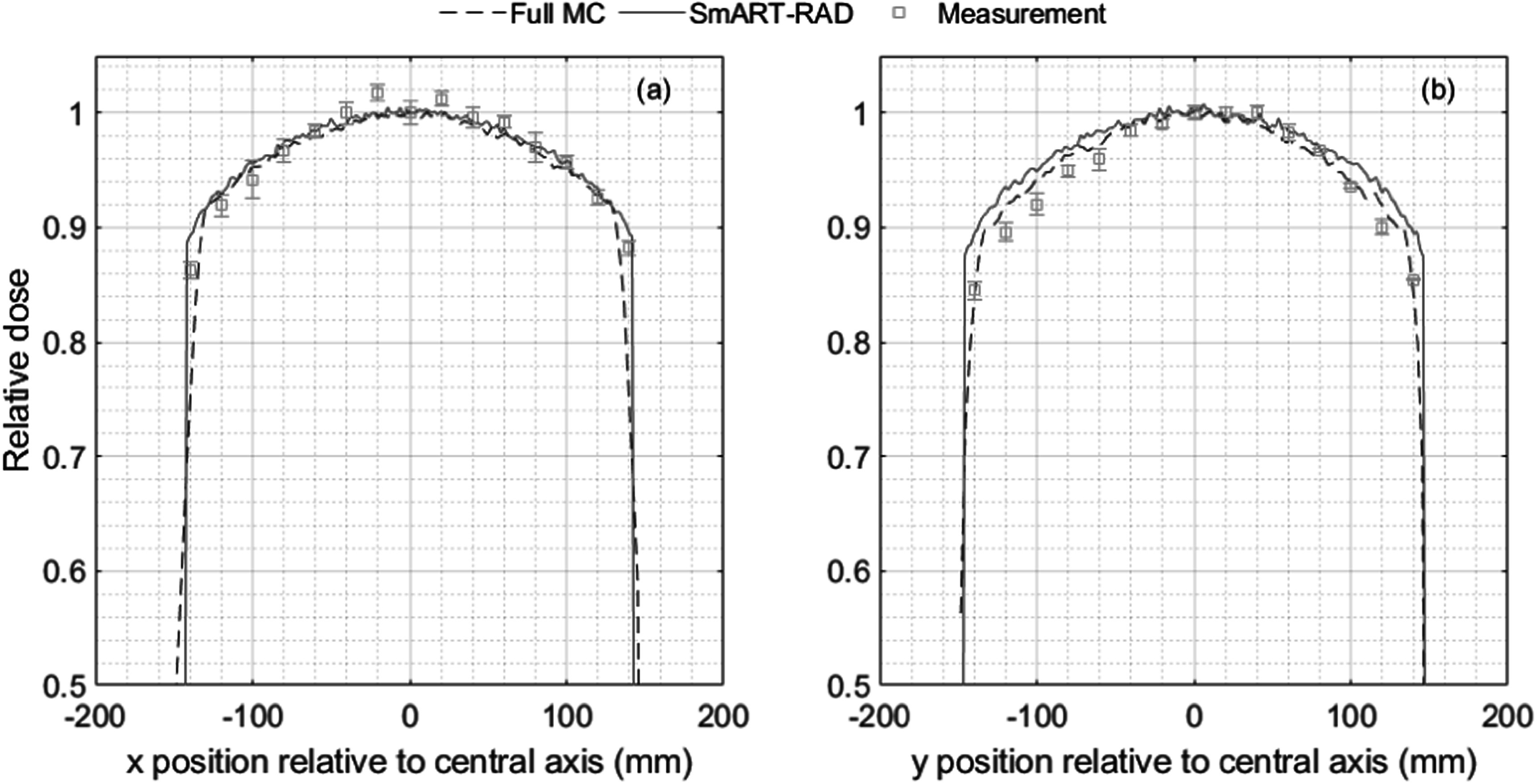
Variation in radial dose across the shelf for 300 kV x-rays. (A) Measured and simulated (with SmART-RAD and full MC) radial variation in the *x*-direction (perpendicular to the tube) (b) in the *y*-direction (parallel to the tube, with positive numbers for the *y*-axis corresponding to the direction of the anode) across the centre of the shelf.

The measured and SmART-RAD simulated variation in radial dose across the shelf for an uncollimated beam for 300 kV x-rays is shown in figure 5. SmART-RAD uses a point source therefore deviations in radial dose can be expected, an additional MC simulation (Full MC) was performed where a phase space was scored using BEAMnrc to include additional effects such as anode heel effect, scattering in the filter and primary collimator. This was done to see if a more complete physics approach would result in better agreement with measurements. There is good agreement with measurements over the middle 100 mm region, but the falloff with radial distance is more pronounced in the measured distribution compared to the simulated values near the outer regions of the beam, the full MC shows an improvement in following measured dose at larger radial distances in the *y*-direction. With the analytical correction on the point source, as described in section 2.6, the agreement in the *x*-direction between the full source simulation from −130 to 130 mm was within 2.1% for all voxel positions with a mean difference of 0.4%. For the *y* direction differences were larger but still within 3.1% with a mean difference of 1.2%. The difference in *y*-direction will be larger with a smaller anode angle and a lower kV value because of the anode heel effect. The agreement between measurement and SmART-RAD in the *x*-direction was within 4.7% for all positions with a mean difference of 0.7%. The *y*-direction showed larger differences up to within 7.2% for the *y*-direction and a mean difference of 2.3%.

## 4. Discussion

The results illustrate the usefulness of using MC simulations to go from a limited number of dosimetry measurements to detailed 3D dose distributions through an irradiated sample. A comparison between the measured values of air kerma and the analytically derived values (table 3) are relatively constant across the three energies used. From this it can be concluded that the ratio of the integrals of two analytically determined spectra (particles/mAs) can be used to scale the MC simulation units (Gy/particle) with a single experimental reference measurement, enabling the simulation to calculate absolute dose (Gy). The agreement between the SpekCalc and measured air kerma can be improved by fine tuning the Nf and P parameters provided by SpekCalc. These parameters are intended to scale the spectrum for a specific x-ray tube but as the internal scaling resulting from these parameters is linear for the bremsstrahlung and characteristic x-rays parts of the spectrum, respectively, this would not impact the ratios and stability between different spectra and were therefore not modified in this study.

The simulated and experimentally determined depth dose distributions for plastic water LR showed very good agreement (figure 3(a)) with a maximum deviation of less than 2%. Likewise, the simulation of radiochromic film between slabs of RB2, bone-equivalent material, also gives good agreement (figure 3(b)), with deviations only drifting above 2% at a depth of 20 mm, with slightly greater deviations for the lower energies. In addition, MC provides the dose to the bone regions (dose-to-bone-in-bone). There is generally good dosimetric agreement in the irradiated area of the centred slit exposures. However, the experimental data observed a larger measured dose in the shielded/non-irradiated area compared to the calculated dose; this difference is on average approximately 5% compared to the maximum dose, irrelevant for most studies but this limitation should be taken into account when a study needs very accurate out-of-field dose values. These differences in out-of-field dose result from the limited amount of backscatter scatter from the table further away from the target geometry not modelled during simulation. SmART-RAD crops the geometry to a limited region around the geometry of interest to reduce calculation time of particles that would miss the area of interest. This backscatter further away from the main beam will have a minimal impact on the main irradiated area but will result in large relative differences in the shielded areas. Optionally you can turn on the full table in SmART-RAD which improves agreement in shielded regions at the cost of an increased calculation time, the results of this difference are shown in figures S2 to S3. The exposures with the phantom at 100 mm offset from the centre showed a larger dosimetric disagreement, as simulation seemed to overestimate the dose. This is consistent with the variation in radial dose distribution in a large open field shown in figure 5, which shows that at lateral distances beyond the 100 mm region, the falloff with radial distance is more pronounced in the measured distribution compared to the simulated values. While analytical corrections may help improving the radial dose drop-off it could be further improved by using more recent x-ray spectrum calculation models to account for the anode heel effect such as SpekPy v2 (Poludniowski *et al* 2021).

Good spatial agreement is seen in the penumbra regions: for situations where lead is used to restrict the radiation field the simulations tend to overestimate the beam size compared to films, but differences are within 0.2 mm on average and never exceed 0.5 mm which is acceptable for most applications in non-image guided cabinets. This overestimation may be an effect of the voxelization of the geometry. While the spatial agreement is acceptable, there is also a slight discrepancy due to uncertainty in the alignment of the x-ray tube and associated x-ray beam to the centre of the shelf, which would require slight optimisation of the simulation geometry if greater spatial accuracy perpendicularly to the beam is required.

The focus of this study was to evaluate the relative agreement between measurements and SmART-RAD within the capabilities available in SmART-RAD. SmART-RAD at present does not have the capability of inserting rectilinear films (active and passive layers) into the geometry overriding previous values therefore H_2_O layers were chosen. This potentially introduces additional uncertainty to the measurement over scoring dose to water in film with the materials defined to the exact EBT3 composition. This effect is most important for films used in a parallel orientation there is better agreement between the percentage depth dose (PDD) in water and film in perpendicular orientation. Robinson *et al* cites a maximum deviation of 5.7% difference between water and film in a perpendicular orientation but in that study many more films over larger depths are taken therefore it can be expected that the cumulative effect is smaller on our data (Robinson *et al* 2020).

The SmART-RAD MC modelling software is written as an accessible tool for most users of x-ray and *γ*-ray sources enabling them to calculate dose distributions through irradiated samples giving good reproducibility to experimental data with a minimal amount of input data. This not only useful for planning irradiations but also to determine dose and dose distributions in a sample which may be difficult to measure directly. However, if greater accuracy is required then the code could be modified by replacing the analytic spectrum and the point source with a full phase space source (as in figure 5). This would have the advantage of dealing directly with the heel effect and its associated radial distributions (currently handled analytically), modelling of focal spot heterogeneities and more accurate modelling of the geometric penumbra near beam edges and collimators. In the present work, the good agreement between simulations and measurements, indicates a sufficient accuracy has been achieved with SmART-RAD.

**Figure 6.**
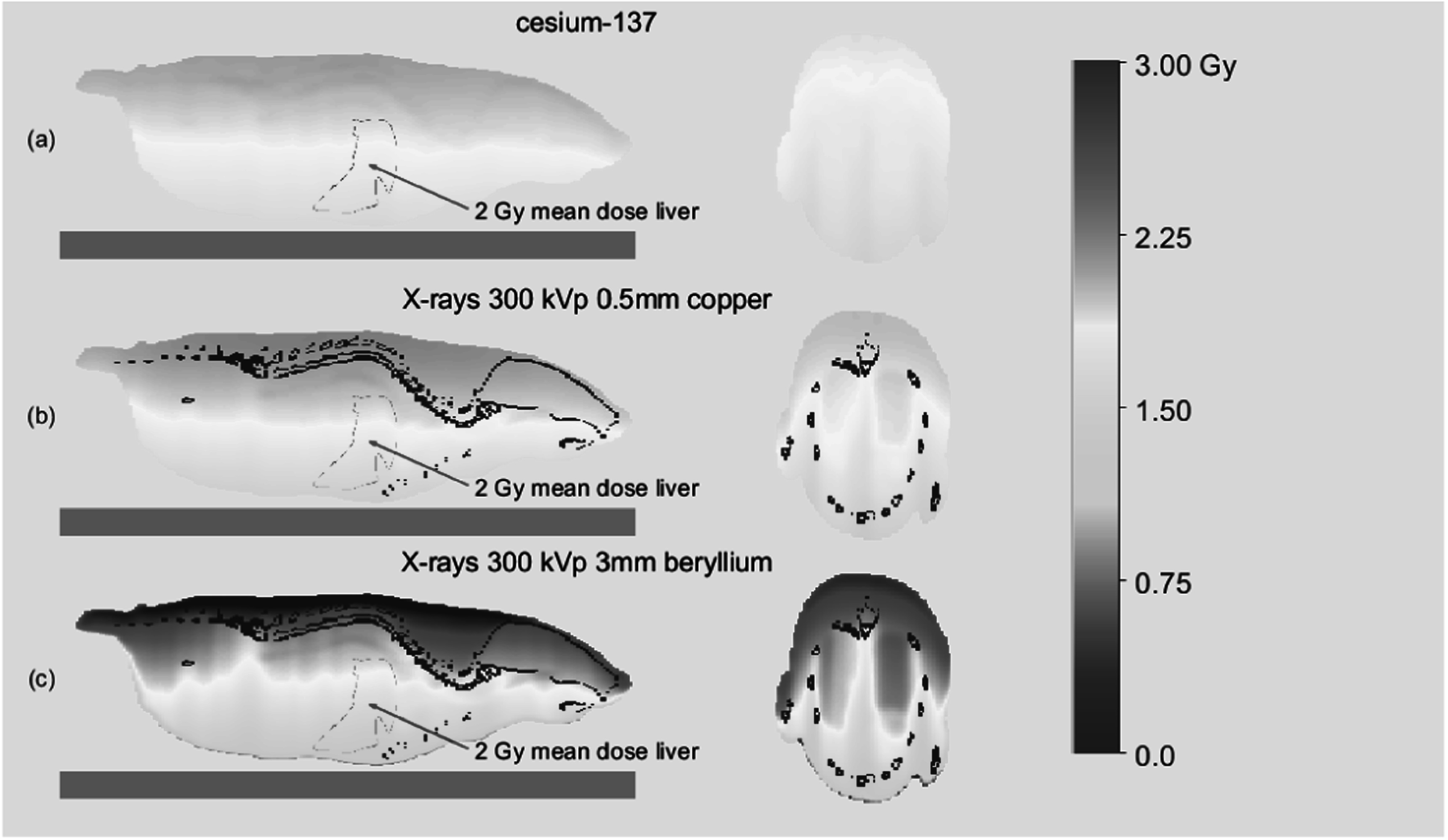
Illustration of the heterogeneity of dose distributions at different energies and filtrations in a mathematical mouse model (MOBY) with 0.3 mm resolution in sagittal and axial views (Segars *et al* 2004). Simulations were performed in SmART-RAD with a target mean dose of 2 Gy to the liver for (a) Cs-137, (b) 300 kVp x-rays with an inherent 3 mm Be and additional 0.5 mm copper filter and (c) 300 kVp X-rays with only the inherent 3 mm Be filtration (bare x-ray tube).

Accurate dosimetry is essential for basic and preclinical radiobiological studies in order to ensure that data is appropriately interpreted and reproducible between laboratories. Cabinet irradiators used for these studies are often supplied with just air kerma measurements at different shelf heights, rather than absorbed dose to the sample. However, the 3D distribution of absorbed dose to the sample will depend on the x-ray energy spectrum (kV and filtration dependent) as well as its geometry and composition. This can also be important for *in vitro* irradiations especially if unfiltered x-rays are used, but differences become far more pronounced for the larger dimensions and heterogeneity of *in vivo* samples (Kirkby *et al* 2013) as illustrated in figure 6. So, using two different cabinet irradiators to expose a sample to a given air kerma value can result in very different absorbed dose distributions to the irradiated sample. This is particularly critical for cabinet irradiators used for total-body irradiation (TBI) for bone marrow transplantation (BMT) studies on rodents. Here, accurate dosimetry is key due to the need to sufficiently deplete the bone marrow cell population while minimising normal tissue toxicity (Zuro *et al* 2021). Interestingly, while caesium *γ*-rays result in a relatively uniform dose distribution across the mouse, dose to the bone and adjacent hematopoietic cells of the bone marrow will be higher and significantly more non-uniform in the x-ray irradiated mouse (Poirier *et al* 2020). However, considering the composition of bone marrow (high adipose content) which will absorb a lower dose compared to its calcium counterpart, there will be steep dose gradients in the marrow tissue due to the short range of the electrons produced in the trabecular bone. As a result, the dose required for biological equivalency between x-rays and *γ*-rays will depend on the spatial location with respect to the bone of the stem and progenitor populations. Investigation of this phenomenon is outside the scope of this paper. It is clear that air kerma does not reflect the actual dose to the sample being irradiated, which is often difficult to measure directly. The advantage of the MC software as described here is that it can be used to calculate absolute 3D dose, based on a single calibration factor for a single x-ray spectrum, determined during commissioning on reference measurements and the geometry and composition of the sample and any associated support or shield. These geometries can range from a petri dish with cells to a heterogeneous specimen like a mouse based on a 3D images (CT/MRI) or using a 3D mathematical phantom (Segars *et al* 2004). Figure 6 shows the dose distribution through a mathematical mouse phantom for three different radiation sources and illustrates the impact of differences in x-ray absorption through bone, adipose, tissue, lung for different photon energy spectra.

While a single calibration factor can be used with an accuracy of 5% on our dataset, if higher accuracy is required a separate air kerma measurement per spectrum could be used to determine a *S*_ref_ resulting in a unique calibration factor per spectrum. When multiple *S*_ref_ are available but not for the selected spectrum, SmART-RAD will scale from the *S*_ref_ that has the closest matching spectrum (as a function of half value layers (HVL)). MC modelling can also be useful for modelling interface effects, for example *in vitro* irradiations are sometimes performed with cells grown as an attached monolayer on glass (often used in conjunction with immunofluorescence staining), rather than plastic, and this results in an increase in dose to the cells from backscattered photons and electrons due to the increased absorption of x-rays in the glass (with its higher effective atomic number compared to the cell culture medium). For larger specimens such higher cross-section for photoelectric effect in backscatter material can then also reduce dose at larger distances from the interface due to reduced Compton backscatter (Verhaegen and Seuntjens, 1995). While this effect is difficult to directly measure or account for with simple point dose calculations, MC simulations can be used to calculate the difference in dose to cells grown on glass compared to plastic as well as determine the changes of dose throughout a larger specimen as a result of changing backscatter material and thickness.

Rather than just relying on air kerma measurements, it is beneficial to also perform dosimetry measurements in a phantom. These can either be a simple geometric phantom made from water or tissue equivalent material (e.g. 2.0 × 2.5 × 6.5 cm^3^ PMMA mouse phantom (Zoetelief *et al* 2001)) which has the advantage of simplicity and can easily be replicated between labs, or more anatomically correct phantoms (Van Hoof *et al* 2013, Soultanidis *et al* 2019). While these more anatomically correct phantoms may better represent the dose heterogeneity through a rodent, they are more difficult to replicate between laboratories, and the measured dose rates will be more variable across the phantom. MC simulations with SmART-RAD also provide the ability to model these dosimetry phantoms, including any dosimeters they may contain. SmART-RAD is intended to be used to plan irradiations in advance as the MC simulations require several minutes of calculation time to provide good statistics, there is an option to have fast calculations of only several seconds that provide a good estimation of the dose with a higher uncertainty, users who are MC experts can modify all MC related parameters to optimize their use cases potentially improving efficiency and thus speed of calculations over the default settings. Finally, having more accurate dose distributions may contribute towards refining and reducing animal experimentation.

## 5. Conclusion

We demonstrate that simulations performed with SmART-RAD gave very good agreement with dose measurements for the depth dose distributions in both water and bone equivalent materials, and good agreement with measured 3D dose distributions. These MC simulations therefore provide a useful tool to go from a single or a limited number of dosimetry measurements to detailed 3D dose distributions through an irradiated sample. This is particularly important when trying to determine dose to particular organs/targets within irradiated rodents. As a result this not only enables an improvement in the accuracy of delivery and reporting of relevant doses, but potentially can be used to retrospectively analyse experimental studies in the literature for much more meaningful comparisons between experiments and laboratories. SmART-RAD may also be an aid to support the current switch from Cs irradiators to kV x-ray irradiators.

## Data Availability

All data that support the findings of this study are included within the article (and any supplementary information files).
